# The Recovery of Neurological Function and Walking Ability After Total En Bloc Spondylectomy for Spinal Tumors

**DOI:** 10.7759/cureus.80675

**Published:** 2025-03-16

**Authors:** Yuki Kurokawa, Satoshi Kato, Noriaki Yokogawa, Takaki Shimizu, Hideki Murakami, Satoru Demura

**Affiliations:** 1 Department of Health Sciences, Nagoya City University, Nagoya, JPN; 2 Department of Orthopaedic Surgery, Graduate School of Medical Sciences, Kanazawa University, Kanazawa, JPN; 3 Department of Orthopaedic Surgery, Graduate School of Medical Sciences, Nagoya City University, Nagoya, JPN

**Keywords:** ambulation, neurological function, spine tumor, total en bloc spondylectomy, walking ability

## Abstract

Background and objective

Total en bloc spondylectomy (TES) is an excision surgery for spinal tumors. TES is a procedure that requires tremendous skills, and hence it is associated with a potential risk of neurological complications. This study aimed to examine the incidence of postoperative neurological dysfunction and to evaluate the recovery of neurological outcomes and walking ability after TES.

Methods

We identified 71 patients who underwent TES for primary and metastatic spine tumors between 2010 and 2017. Perioperative neurological function and ambulation status were evaluated preoperatively, at one week, and one, three, and six months postoperatively.

Results

Postoperative neurological deficits were observed in 26 patients (37%). In patients with preoperative neurological deficits, an improved modified Frankel grade was observed up to six months (22/31, 71%). Six months after the surgery, the ambulation rate was significantly higher compared to that before surgery. All 14 of 40 patients (35%) with no neurological deficits preoperatively who had neurological deficits postoperatively recovered the neurological function and were able to walk at three months postoperatively.

Conclusions

Although some patients in our cohort showed postoperative neurological deterioration after TES, their neurological function and walking ability continued to improve over a prolonged period.

## Introduction

Total en bloc spondylectomy (TES) is one of the surgical procedures employed in the treatment of spinal tumors. TES enables complete resection of the diseased vertebrae to achieve local control [1-3]. Recent studies have demonstrated that spinal metastasectomy of certain kinds of carcinoma such as kidney and thyroid could potentially prolong survival in selected patients [4,5]. However, TES is a technically challenging surgical procedure, which is associated with a high risk of perioperative systemic complications rate [6], resulting in deterioration in activities of daily living (ADLs) [7,8]. In particular, postoperative neurological deficits and gait disturbances may significantly reduce a patient’s ADLs. Very few studies have reported improvement in neurological function after surgery for spinal tumors in patients with preoperative neurological deficits [9,10]. In addition, the incidence of neurological deficits, gait disturbances, and recovery of neurological outcomes after TES has not been previously investigated. This study aimed to examine the incidence of postoperative neurological dysfunction and to evaluate the recovery of neurological outcomes and walking ability after TES.

This article was previously presented as a meeting abstract at the 2023 American Academy of Orthopaedic Surgeons Annual Meeting.

## Materials and methods

Ethical approval

This study was approved by the Ethics Committee of Kanazawa University Hospital [approval no: 2015-077 (1895)], and informed consent was obtained from all study participants and/or their guardians for including their data.

Study sample

This study was conducted from September 1, 2015 to March 31, 2020. We included patients who underwent TES for primary and metastatic spine tumors between 2010 and 2017 and for whom data of detailed neurological symptoms could be collected up to six months postoperatively; we excluded patients who underwent surgery other than TES for spinal tumors or whose data could not be followed up to six months postoperatively. We identified 71 patients (45 males and 26 females) with a mean age of 54.7 years at the time of surgery. Of them, 15 had primary malignant tumors (leiomyosarcoma in three, chordoma in three, chondrosarcoma in two, and others in seven), 14 had aggressive benign tumors (giant cell tumor in seven, aggressive hemangioma in seven), and 42 had metastatic tumors (kidney in 29, thyroid in 13). The tumor was located in the thoracic spine in 49 patients, and the lumbar spine in 22. The surgical indication for metastatic tumors was determined based on the patient’s choice and the following criteria: stable disease with no other or a limited number of metastases, operability (Karnofsky Performance Scale ≥30% or Eastern Cooperative Oncology Group performance status ≤3), and surgical feasibility (a tumor involving ≤3 consecutive spinal vertebrae).

Surgical procedure

Our surgical technique for TES has been described in detail elsewhere [3]. The technique consists of two steps including en bloc resection of the posterior element and en bloc resection of the anterior element, maintaining the function of the spinal cord. For TES in the thoracic spine, a single posterior approach is employed, with the transection of the bilateral nerve roots at the tumor level for the resection of the vertebral body. In some patients with large tumors that expand anteriorly to the paravertebral area, additional dissection via an anterior approach was required. In the lower lumbar spine, a combined approach was applied because the nerve roots at the tumor level must be preserved for postoperative lower limb function. The anterior column was reconstructed by inserting a vertebral spacer packed with an autograft. In all cases, posterior instrumentation was performed. 

Data evaluation

Patient data were extracted from their medical charts. Perioperative complications within two months of surgery, such as deep surgical site infection, cerebrospinal fluid leakage, and respiratory and cardiovascular complications, were recorded. Neurological function was evaluated preoperatively using the modified Frankel grade (Table 1) [11] and at one week, and one, three, and six months postoperatively. Postoperative neurological deficits were defined as changes in at least one modified Frankel grade compared to preoperatively, and the rate of postoperative neurological change was also evaluated. Ambulatory rate was defined as the percentage of patients with the ability to walk without walking aids or with a cane or walker and was compared preoperatively, and at one week, and one, three, and six months postoperatively. 

**Table 1:** Modified Frankel grade Neurological function was assessed preoperatively by using the modified Frankel grade

Grade	Neurological status
A	Complete motor and sensory loss
B	Preserved sensation only, voluntary motor function absent
C	Preserved motor loss than fair grade (nonfunctional for any useful purpose)
D1	Preserved motor at lowest functional grade (3+/5+) and/or with bowel or bladder dysfunction
D2	Preserved motor at mid functional grade (3+ to 4+/5+) and/or neurologic bowl or bladder function
D3	Preserved motor at high-function grade (4+ to 5+) and normal voluntary bowel or bladder function
E	Complete motor and sensory function normal (may still have abnormal reflexes)

Statistical analysis

Countable data were expressed as percentages; comparisons between groups were performed using the chi-square test or Fisher’s exact tests. Continuous variables were expressed as means and standard deviation (SD) for parametric data and a t-test was used to compare between groups. The medians [interquartile range (IQR)] were used for nonparametric data. The Mann-Whitney U test and Wilcoxon matched-paired singed-rank test were used for comparisons. Statistical significance was set at p<0.05. All statistical analyses were performed using the JMP version 11 software program (SAS, Cary, NC).

## Results

Incidence of postoperative neurological deficits

Before surgery, 40 patients had a neurologically normal (modified Frankel grade E) status. Preoperative neurological deficits were found in 31 patients (grade A in one, B in two, C in nine, D1 in two, D2 in four, and D3 in 13 patients). Ambulatory rates were 55% with preoperative neurological deficits and 100% without preoperative neurological deficits, respectively (p<0.01) (Table 2). 

**Table 2:** Comparison of patient’s characteristics based on preoperative neurological deficit *P<0.05. **P<0.01 SD: standard deviation

Variables	Total (n=71)	Preoperative neurological deficits		P-value
		Yes (n=31)	No (n=40)	
Mean age (SD)	54.7 (13.3)	54.4 (13.9)	54.8 (12.9)	0.88
Sex (male: female)	45:26	18:13	27:13	0.46
Preoperative modified Frankel grade, n				
A	1	1	0	-
B	2	2	0	-
C	9	9	0	-
D1	2	2	0	-
D2	4	4	0	-
D3	13	13	0	-
E	40		40	-
Diabetes, n	9	6	3	0.16
Tumor type, n				
Aggressive benign	14	6	8	-
Primary malignant	13	7	6	-
Metastatic	42	16	26	-
Primary unknown	2	2	0	-
Tumor location, n				
Thoracic spine (T1 to T12)	49	22	27	-
Lumbar spine (L1 to L5)	22	9	13	-
Preoperative radiation therapy, n	12	8	4	0.11
Resected vertebrae				
1	47	17	30	-
2	12	5	7	-
3 or more	12	9	3	-
Mean operative time, minutes (SD)	500.5 (201.1)	550.1 (256.6)	426.1 (135.8)	0.07
Mean intraoperative bleeding, ml (SD)	673.0 (872.7)	898.1 (1253.8)	498.5 (293.6)	0.06
Complications, n (%)				
Respiratory	10 (15.1%)	5 (16.0%)	5 (14.3%)	0.83
Cardiovascular	1 (1.5%)	1 (3.2%)	0 (0%)	0.46
Infection	8 (11.3%)	5 (16.1%)	3 (7.5%)	0.28
Cerebrospinal fluid leakage	10 (14.1%)	8 (25.8%)	2 (5.0%)	0.02*
Neurological deficit	26 (36.6%)	12 (38.7%)	14 (35.0%)	0.80
Preoperative ambulation rate, n (%)	57 (80.3%)	17 (54.8%)	40 (100%)	<0.01**

Postoperative neurological deficits were observed in 26 patients (37%). Among them, 14 (54%) showed no preoperative neurological deficits. There was no significant difference in the incidence of neurological deficits preoperatively and postoperatively. The incidence of neurological deficits was significantly higher in patients who underwent TES in the lumbar spine (18/22, 82%) than in those who underwent in the thoracic spine (9/49, 18%) (p<0.01). Among the 14 patients without preoperative neurological deficits but developed postoperative neurological deficits, 11 patients (79%) underwent TES in the lumbar spine.

Recovery of neurological function and ambulatory status

**Figure 1:**
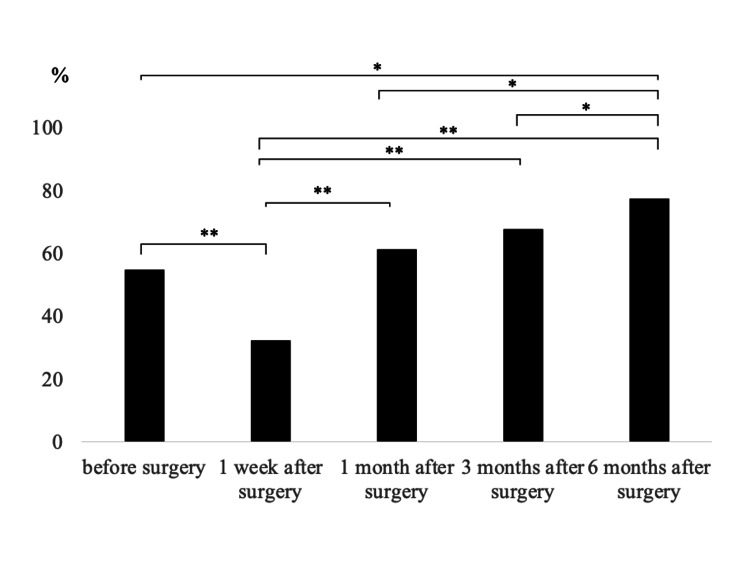
The ambulation rate in patients with preoperative neurological deficit *P<0.05. **P<0.01

Table 3 shows the modified Frankel grade in patients with neurological deficits before surgery. Among patients with preoperative neurological deficits, improvement in the modified Frankel grade was observed in six patients (19%) at one week, 14 patients (45%) at one month, 11 patients (36%) at three months, and in eight patients (26%) at six months postoperatively. Ten patients (32%) were able to walk without aids or by cane or walker at one week, 19 patients (61%) at one month, 21 patients (68%) at three months, and 24 patients (77%) at six months after TES. Ambulatory rates were significantly lower than at one week postoperatively but continued to improve significantly compared to one week postoperatively until one (p<0.01), three (p<0.01), and six months (p<0.01) postoperatively. Six months after surgery, the ambulatory rate was significantly higher than before surgery (p<0.05), one month (p<0.05), and three months (p<0.05) after surgery (Figure 1). Of the 14 patients unable to walk before surgery, five, two, and three patients regained the ability to walk one, three, and six months after surgery, respectively.

**Table 3:** Modified Frankel grade in patients who showed preoperative neurological deficit (n=31)

Modified Frankel grade	Preoperative	1 week	1 month	3 months	6 months
A	1	3	3	3	3
B	2	1	0	0	0
C	9	9	8	1	1
D1	2	8	2	6	3
D2	4	4	8	6	5
D3	13	5	9	14	16
E	0	1	1	1	3

**Figure 2:**
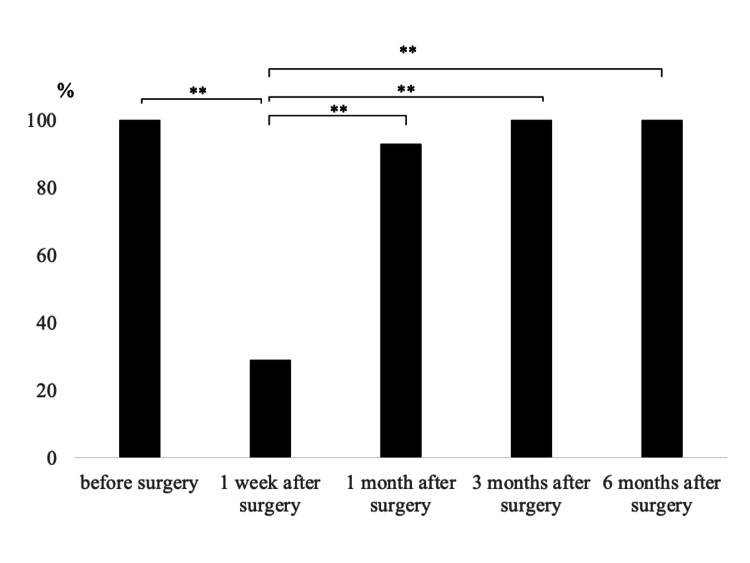
The ambulation rate in patient with postoperative neurological deficit who had no neurological deficit before surgery *P<0.05. **P<0.01

Table 4 shows the modified Frankel grade in patients with preoperative modified Frankel grade E who developed neurological deficits postoperatively (n=14). Among these 14 patients, improvement in the modified Frankel grade was observed in 11 patients (79%) at one month, in six patients (43%) at three months, and in no patients (0%) at six months postoperatively. Four (29%) patients were able to walk without aids or by cane or walker at one week, 13 patients (93%) at one month, 14 patients (100%) at three months, and 14 patients (100%) at six months postoperatively. Compared to preoperative values, ambulatory rates were significantly lower than at 1 week postoperatively, and they continued to improve significantly compared to one week postoperatively until one (p<0.01), three (p<0.01), and six months (p<0.01) postoperatively (Figure 2). Patients who were unable to walk one week postoperatively regained the ability three months after the surgery.

**Table 4:** Modified Frankel grade in patients with no preoperative neurological deficit but who developed neurological deficits postoperatively (n=14)

Modified Frankel grade	Preoperative	1 week	1 month	3 months	6 months
C	0	1	0	0	0
D1	0	9	1	0	0
D2	0	2	5	3	3
D3	0	2	8	9	9
E	14	0	0	2	2

## Discussion

The incidence of perioperative complications after TES is approximately 40% [7, 12], with a reported incidence of 11% for neurological deficits [13]. In this study, the incidence of neurological deficits after TES was 37% (26/71 patients), which is higher compared to that in previous studies. Most patients in our study who had postoperative neurological deficits had undergone lumbar TES (18/26 patients, 69%).

Lumbar TES is technically challenging because of the local anatomy. Compared to thoracic TES, lumbar TES usually necessitates extensive nerve root dissection with frequent retraction from the tumor because of the size of the lumbar spine and lumbosacral plexus [14]. Shimizu et al. reported that 87% of patients who underwent lumbar TES developed at least one perioperative complication, with the most common issue being lower extremity muscle weakness [8]. Tang et al. reported that lumbar location, involved vertebrae, and combined approach as predictors of preoperative neurological deficits after TES [15]. In the present study, most of the patients with postoperative neurological deficits had undergone lumbar TES. Patients who underwent thoracic TES included those with more than three vertebral resections and those who had undergone a combined anterior-posterior approach.

Recovery of neurological function and ambulation varied according to preoperative neurological deficits. In patients with preoperative neurological deficits, 19% (6/31 patients) showed an improvement of 1 or more grades in the modified Frankel grade at one week postoperatively, and improvement was observed up to six months postoperatively. Recovery of walking ability showed a similar process, with 77% (24/31 patients) being able to walk at six months postoperatively, significantly improving preoperative walking ability. Among the patients who regained the ability to walk postoperatively, 10 could not walk at all before surgery. Xu et al. performed TES on patients with preoperative paralysis and reported that 92% had good motor function at three months postoperatively [16].

Liu et al. reported that almost all patients showed improvement in neurological symptoms six months after TES, while the recovery rate of walking ability in patients who could not walk preoperatively was 45%[17]. Compared to the previous study, the present study showed a higher recovery rate of walking ability in patients who were unable to walk preoperatively. However, four patients who were unable to walk preoperatively did not regain their walking ability up to six months postoperatively. These patients had preoperative modified Frankel grade A and postoperative neurological deficits resulting in modified Frankel grade A. In a previous study, two-thirds of Frankel grade A patients showed neurological recovery after TES. However, one patient did not recover [18]. The challenges related to postoperative neurological symptoms and walking ability in Frankel A patients were indicated. 

Patients without preoperative neurological deficits showed faster postoperative neurological deficits recovery compared to those with preoperative neurological symptoms. Most patients underwent lumbar TES (11/14, 79%), and although 10 were unable to walk in the first postoperative week, all were ambulatory at three months postoperatively. Recovery of neurological symptoms showed a similar trend. While lumbar TES is associated with a high incidence of postoperative lower extremity muscle weakness, a good prognosis for recovery has been reported [8]. Although three patients who underwent thoracic TES were included among those with postoperative neurological deficits, all patients were able to achieve walking ability. The results of this study suggest that patients without preoperative neurological deficits have a higher likelihood of regaining their ability to walk, even if a temporary loss of walking ability occurs postoperatively, compared to those with preoperative neurological deficits. Furthermore, although patients with preoperative neurological deficits showed long-term recovery of neurological symptoms and walking ability for up to six months postoperatively, they required more time to recover than patients without preoperative neurological deficits.

The limitations of the present study include the small and heterogeneous cohort and its single-center, retrospective design. Furthermore, we could not clarify the long-term neurological recovery and walking ability for more than six months after surgery. In addition, postoperative pain assessment was not assessed in this study. As TES is a highly invasive procedure, postoperative pain may have influenced muscle weakness in the lower extremities. Also, we had not adequately tested the intra-examiner reliability of the modified Frankel classification. Furthermore, the reproducibility may be inadequate because missing data imputation was not performed in this study. Despite these limitations, the present study is one of the few reports showing a specific course of recovery of neurological deficits and walking ability after TES. In addition, this study is based on data from a physiotherapist’s detailed assessment of neurological symptoms, which may be more accurate than previous studies where physicians assessed patients retrospectively.

## Conclusions

Our results showed postoperative neurological deficits in 37% of the cohort, with a significantly higher incidence in patients who underwent lumbar TES. Despite these deficits, a significant proportion of patients experienced neurological recovery and regained ambulation within three months postoperatively. In particular, patients without preoperative neurological deficits demonstrated rapid recovery, with all patients who developed transient neurological deficits after surgery regaining ambulation within three months. Patients with preoperative neurological deficits also showed improvement, with walking ability continuing to improve over six months, although recovery took longer in this group. The results highlight the potential for neurological recovery and functional improvement after TES, even in patients who experience transient neurological deterioration. Although the incidence of postoperative complications was higher for lumbar TES, the overall prognosis for ambulation was favorable, with most patients regaining ambulation within months. However, Frankel grade A patients had a more difficult recovery and may require longer rehabilitation. Our findings provide valuable insights into the recovery process of patients undergoing TES for spinal tumors. Further research with larger and more diverse cohorts, as well as long-term follow-up, is needed to better understand the full extent of recovery after TES and to refine postoperative management strategies.
